# ACE2 mouse models: a toolbox for cardiovascular and pulmonary research

**DOI:** 10.1038/s41467-020-18880-0

**Published:** 2020-10-14

**Authors:** Hongpeng Jia, Xinping Yue, Eric Lazartigues

**Affiliations:** 1 Division of Pediatric Surgery, Department of Surgery, Johns Hopkins University School of Medicine, Baltimore, MD 21205 USA; 2 Department of Physiology, Louisiana State University Health Sciences Center, New Orleans, LA 70112 USA; 3 Department of Pharmacology & Experimental Therapeutics, Louisiana State University Health Sciences Center, New Orleans, LA 70112 USA; 4 Cardiovascular Center of Excellence, Louisiana State University Health Sciences Center, New Orleans, LA 70112 USA; 5 Southeast Louisiana Veterans Health Care Systems, New Orleans, LA 70119 USA

**Keywords:** Genetic engineering, Infection, Viral infection, Experimental models of disease

## Abstract

Angiotensin-converting enzyme 2 (ACE2) has been identified as the host entry receptor for the severe acute respiratory syndrome coronavirus 2 (SARS-CoV-2) responsible for the COVID-19 pandemic. ACE2 is a regulatory enzyme of the renin-angiotensin system and has protective functions in many cardiovascular, pulmonary and metabolic diseases. This review summarizes available murine models with systemic or organ-specific deletion of ACE2, or with overexpression of murine or human ACE2. The purpose of this review is to provide researchers with the genetic tools available for further understanding of ACE2 biology and for the investigation of ACE2 in the pathogenesis and treatment of COVID-19.

## Abstract (web summary)

Angiotensin-converting enzyme 2 (ACE2) is a cell surface enzyme previously shown to mediate SARS-CoV, and now SARS-CoV-2, entry into host cells. Here the authors review existing mouse ACE2 models expressing humanized, transgenic, knockout, knockin, conditional and reporter alleles to provide a toolbox for COVID-19 research.

## Introduction

**Fig. 1:**
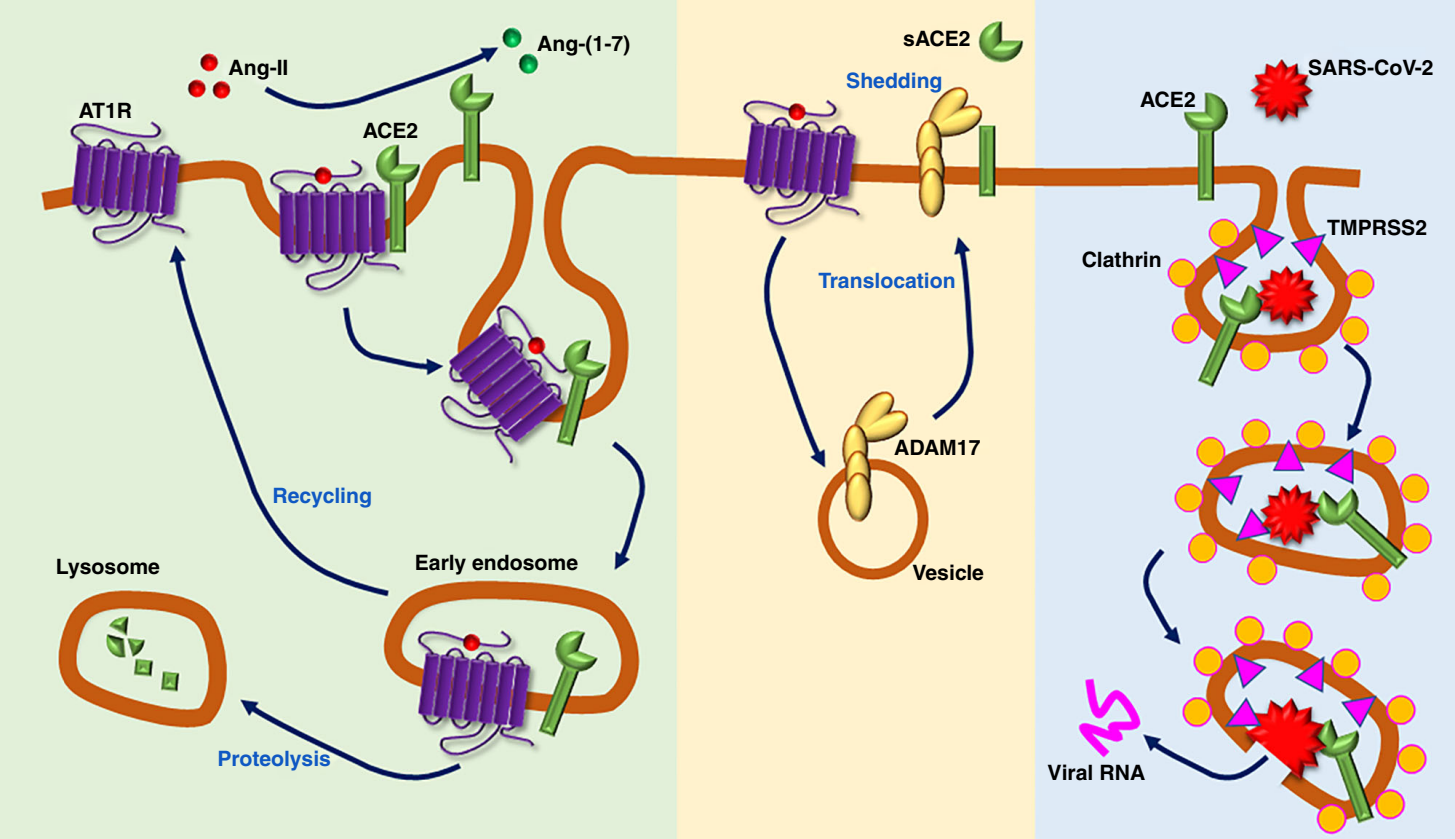
Mechanisms of ACE2 post-translational regulation and viral infection. Angiotensin (Ang)-II binding to its type 1 receptor (AT_1_R) leads to internalization of the AT_1_R–ACE2 complex to early endosomes, thus preventing further Ang-II hydrolysis into Ang-(1–7). AT_1_R is then recycled to the plasma membrane, while ACE2 is degraded in lysosomes (left). In addition, AT_1_R activation by Ang-II leads to the recruitment of ADAM17 from cytoplasmic pools and translocation to the plasma membrane, where it sheds the ACE2 ectodomain to release a secreted form of the enzyme (sACE2) (middle). Finally, ACE2 serves as the receptor for SARS-CoV-2, triggering endocytosis via clathrin-coated pits. Spike protein priming by serine protease TMPRSS2 is also required for viral entry (right).

Angiotensin-converting enzyme 2 (ACE2) was originally discovered in 2000 as a monocarboxypeptidase capable of cleaving Angiotensin (Ang)-II into the shorter Ang-(1–7) peptide^1,2^. Additional substrates of ACE2 were later shown to include des-Arg^9^-bradykinin, Apelin 13 and 36, β-casomorphin, neocasomorphin, and dynorphin A^3^. The ACE2 gene is located on the X chromosome and contains 18 coding exons in humans (19 in mice), among which exon 9 hosts a single zinc-binding domain (HEMGH), the siege of enzymatic activity^4^. Interestingly, while ACE2 expression is largely driven by a proximal promoter in most tissues, it is controlled in the lung by a distal promoter^5^. Most of the initial interest in ACE2 revolved around the renin–angiotensin system and ACE2’s ability to counter overactivity of this system, notably in cardiovascular and pulmonary diseases. Indeed, by metabolizing the octapeptide Ang-II to Ang-(1–7), ACE2 reduces the activation of the Ang-II type 1 receptor (AT_1_R) responsible for sympatho-excitation, salt and water reabsorption, aldosterone secretion, vasopressin release, and vasoconstriction (Fig. 1). ACE2 expression was found to be beneficial for many diseases, including systemic hypertension, pulmonary hypertension (PH), heart failure, diabetes, kidney injury and liver fibrosis, among others. However, post-translational mechanisms such as disintegrin and metalloproteinase domain-containing protein 17 (ADAM17)-mediated shedding^6,7^ and AT_1_R-dependent internalization^8^ have been shown to limit the beneficial effects of ACE2 (Fig. 1). To overcome these limitations, research groups investigated the potential therapeutic benefits of increasing ACE2 expression or activity using recombinant human ACE2, gene therapy, or ACE2 “*activators*”^9^. Unexpectedly, ACE2 was identified as a *receptor* for various coronaviruses including the severe acute respiratory syndrome coronavirus (SARS-CoV)^10^ during the SARS epidemic that spread through China and 25 other countries in 2002–2003. ACE2 was subsequently reported to be also the *receptor* for NL63 (HCoV-NL63)^11^, as well as SARS-CoV-2, which is responsible for the coronavirus disease of 2019 (COVID-19) pandemic (Fig. 1)^12^. This finding has resulted in a significant amount of studies investigating the SARS-ACE2 relationship, with a battery of murine genetic models being generated in the last two decades to understand the role of ACE2 in cardiovascular regulation and in infectious diseases (Tables 1–3). Because the mouse ACE2 does not bind efficiently to SARS-CoV or SARS-CoV-2^13^, several ACE2-humanized mouse lines have been generated, and can be relevant to research related to the COVID-19 outbreak (Table 4). Unfortunately, not all models have been made available to the research community and identifying a specific model for particular type of studies is often difficult due to the lack of details provided in the original description. In this review, we highlight the different knockout (KO), knockin (KI), conditional KO, and humanized ACE2 mouse models, their primary phenotypes, and their potential applications for ACE2 and coronavirus research.

**Table 1:** Global ACE2 KO models and associated phenotypes.

Country of origin	Phenotypes	Source
Canada	*Cardiovascular:* Enhanced susceptibility to Ang-II or pressure overload-induced heart failure^23,24^, cardiac hypertrophy and myocardial fibrosis^14^, myocardial infarction^25^, vascular dysfunction and atherosclerosis^26,27^, abdominal aortic aneurysm^28^ *Metabolic:* Lipodystrophy and steatosis^29^, obesity-induced epicardial adipose tissue inflammation and insulin resistance^31^, diabetic cardiovascular complications^32^, reduced intestinal uptake of tryptophan and altered serotonin metabolism^37,38^ *Liver:* Hepatic steatosis and fibrosis^29,30^ *Lung:* Resistant to SARS-CoV infection^39^, exacerbated ALI/ARDS due to acid aspiration, lipopolysaccharide or *pseudomonas aeruginosa* infection^41–43^, impaired inactivation of des-Arg^9^-bradykinin^43^, exacerbated lung fibrosis with a sex dichotomy^44^, increased susceptibility to respiratory viral infection^45–47^ *Kidney:* Renal fibrosis and inflammation^33,34^ and diabetes or hypertension associated nephropathy^35,36^	Life Science Institute, The University of British Columbia
USA	**1**. *Developmental:* Gestational impairment in *Ace2*^*−/−*^ dams^50,51^, smaller size and slower to develop^48,49^ *Cardiovascular:* Mild elevation of systolic BP^15^, enhanced Ang-II-induced hypertension^15^ and myocardial dysfunction^52^, diet or diabetes-associated hypertension^48,53^, increased susceptibility to atherosclerotic plaques^55^, autonomic dysfunction and increased oxidative stress^56^, abdominal aortic aneurysms^57^ *Metabolic:* Elevated fasting blood glucose levels^49^, glucose intolerance^49^, and reduced glucose-stimulated insulin secretion and β-cells mass^54^ **2**. *Developmental:* Prenatal lethality, smaller size and reduced weight gain^58^ *Liver:* Enhanced hepatic inflammation and sensitivity to liver fibrosis^58^ *Lung:* Increased inflammatory responses and bronchial hyperplasia when exposed to cigarette smoking^59^ or particulate matter^60^	**1**. Duke University
		**2**. MMRRC (#031665-UCD)
Japan	*Cardiovascular:* Postnatal cardiac hypertrophy^61^, reduction in cardiac contractility and dilatation of the left ventricle in response to pressure overload^17^, increased neointimal formation^63^, atherosclerotic lesions^65^ *Metabolic:* Glucose intolerance and insulin resistance^64^ *Kidney:* Increased baseline Ang-II levels, exaggerated kidney damage in a diabetic model^62^ *Musculoskeletal:* Reduced GLUT4 and MEF2A expression^64^, enhanced expression of sarcopenia-associated genes and lack of muscle strength and bone loss^67^ *Neurological:* Impaired cognitive function with increased AT_1_R and oxidative stress in hippocampus^66^	Osaka University
China	**1**. *Metabolic:* Impaired first phase insulin secretion, enhanced glucose sensitivity^69–71^ *Lung:* Increased lung injury following limb ischemia–reperfusion^73^, exacerbated response to lipopolysaccharide^75^ *Pancreas:* Oxidative stress and dedifferentiated β-cells^69–71^, severe acute pancreatitis^74^ *Kidney:* Increased renal injury following limb ischemia–reperfusion^72^ **2**. Not reported **3**. Increased susceptibility to colitis^21^	**1**. Peking Union Medical College
		**2**. NRCMM/MARC
		**3**. BGI-Shenzhen, China

*MMRRC* Mutant Mouse Resources & Research Centers supported by NIH, *NRCMM/MARC* National Resource Center of Model Mice/Model Animal Research Center of Nanjing University, China. Bold numbers refer to the various models in the order listed in the text.

**Table 2:** Conditional ACE2 KO models and associated phenotypes.

Cell type-specific KO	Phenotypes	Source
Neurons	Reduced inhibitory postsynaptic current of hypothalamic neurons involved in BP regulation^76^	Louisiana State University-New Orleans
Lung epithelium	Greater weight loss, increased neutrophil infiltration, exaggerated lung injury and inflammation in response to bacterial lung infection^42^	Johns Hopkins University
Adipocytes	Augmented systolic BP and acute pressor response to Ang-II injection^77^	University of Kentucky

**Table 3:** ACE2 KI models and associated phenotypes.

ACE2 KI models	Phenotypes	Source
*Ace2* S680D KI	No change in systemic BP, but protective against SU5416/hypoxia + reoxygenation-induced pulmonary hypertension^20^	University of California, San Diego
ROSA26-*Ace2* KI	Reduced anxiety and plasma cortisol levels^80^, protected against myocardial infarction^81^	University of Florida
CRH-*Ace2* KI	Reduced anxiety and plasma cortisol levels^82^	University of Florida
Villin-*Ace2* KI	Personal communication, not yet reported	University of Florida

**Table 4:** Humanized murine ACE2 models and associated phenotypes.

Models	Phenotypes	Source
αMHC-*hACE2*	Low BP, A-V block, and lethal ventricular arrhythmia^83^	Not available due to lethality
K18-*hACE2*	Increased viral titer, exaggerated pulmonary inflammatory response^84,90^, and neuronal cell death following SARS-CoV infection^87^	Jackson Lab #034860
CMV-*hACE2*	Increased viral titer, exaggerated inflammatory response in lungs and brain, and increased mortality following SARS-CoV infection^91,92^	University of Texas Medical Branch
HFH4-*hACE2*	Exaggerated response and increased mortality following SARS-CoV infection^93^	MMRRC #066719-UNC
Syn-*hACE2*	Protected against hypertension^94^ and cardiac hypertrophy^96^ induced by Ang-II infusion, reduced renal sympathetic nerve activity and lower urinary norepinephrine in coronary artery ligation-induced heart failure^95^	Jackson Lab #034899
*mAce2-hACE2*	Increased viral titer and exaggerated pulmonary inflammatory response following both SARS-CoV and SARS-CoV2 infection^97^, reduced renal and lung injury post-hindlimb ischemia-reperfusion^72,73^	Chinese Academy of Medical Sciences/Peking Union Medical College
*hACE2* KI	Not reported	NRCMM/MARC

*MMRRC* Mutant Mouse Resources & Research Centers supported by NIH, *NRCMM/MARC* National Resource Center of Model Mice/Model Animal Research Center of Nanjing University, China.

## Global ACE2 KO models

Following the discovery of ACE2 in 2000, several groups generated mice harboring global deletions of the *Ace2* gene using traditional homologous recombination, transcription activator-like effector nucleases (TALEN) technology, and more recently, CRISPR/Cas9^14–21^. Interestingly, the phenotypes of these mice have not been entirely consistent among laboratories, an issue partly blamed on the genetic background of the models^22^. For convenience, a description of these ACE2 KO models is organized by country of origin (Table 1).

### Canada

**Fig. 2:**
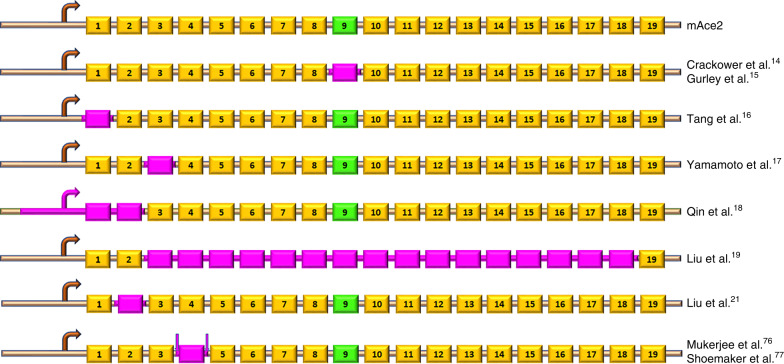
Targeting strategies of the mAce2 gene in KO and conditional KO mice. Schematic of the different ACE2 KO models. The mouse *Ace2* gene contains 19 exons, and the regions deleted in these models are highlighted in purple. Exon 9 (green) codes for the ACE2 active site. The floxed exon is flanked by the LoxP sites.

A widely adopted global ACE2 KO mouse model was generated by Dr. Josef Penninger’s group^14^. The KO mice were made by targeting a 230 bp region containing the zinc-binding catalytic domain with the neomycin resistance gene cassette placed in the anti-sense orientation (Fig. 2). The original genetic background of these KO mice was a mix of C57BL/6 and 129S1/SvImJ. In the initial study using this line, Crackower et al.^14^ reported a severe cardiac phenotype due to the uncoupling of ACE/ACE2 balance. However, after backcrossing onto the C57BL/6 background, the cardiac phenotype was no longer observed^14^. Since then, this line has been widely used in a variety of disease models. Overall, these studies reveal that ACE2 is protective against Ang-II-mediated or pressure overload-induced heart failure^23,24^, myocardial infarction^25^, vascular dysfunction and atherosclerosis^26,27^, abdominal aortic aneurysm^28^, hepatic steatosis and fibrosis^29,30^, obesity or diabetes-associated metabolic and cardiovascular disorders^31,32^, renal fibrosis and inflammation^33,34^, and diabetes or hypertension associated nephropathy^35,36^. Separate from ACE2’s functions in the counterbalance of Ang-II/AT_1_R signaling, studies using this KO line also identified an association between ACE2 and the amino acid transporter B^0^AT1, and deletion of *Ace2* leads to reduced intestinal uptake of tryptophan and altered serotonin metabolism^37,38^. These latter findings are thought to be important in regulation of the microbiome.

Notably, studies using this line to understand the role of ACE2 in acute lung injury (ALI) are particularly relevant to ongoing COVID-19 research. The very first study using this strain of ACE2 KO mice in pulmonary disease settings was reported by Kuba et al.^39^, where the authors infected the KO mice and control counterparts with clinical isolates of SARS-CoV and found that ACE2 KO mice were resistant to SARS-CoV infection. Viral titers from lung tissues of infected ACE2 KO mice were five logs lower than those from SARS-CoV-infected wild-type (WT) mice. Infected ACE2 KO mouse lungs showed no signs of inflammation, whereas most of the SARS-CoV-infected WT mice displayed mild inflammation with leukocyte infiltration. The significance of the study, however, was undermined by the fact that the mild phenotypes observed in WT mice did not reflect the disease severity observed in SARS-CoV patients. In a subsequent study, a mouse-adapted strain of SARS-CoV named MA15 (obtained through 15 passages of SARS-CoV in the respiratory tract of young BALB/c mice in vivo) produced lung and systemic phenotypes comparable to human patients infected with SARS-CoV^40^. A similar approach could be adapted to study COVID-19.

Acute respiratory distress syndrome (ARDS) is a life-threatening form of ALI. Clinical observations from COVID-19 patients indicate that ALI and ARDS are among the prominent factors that cause excessive mortality in the elderly. Several groups have investigated the role of ACE2 in the pathogenesis of ALI/ARDS using the ACE2 KO mice from Penninger’s laboratory. Imai et al.^41^ and our group^42^ revealed that ACE2 KO mice exhibit severe disease progression in comparison to WT mice following acid aspiration-mediated or sepsis and *Pseudomonas aeruginosa* infection-induced ALI/ARDS, as manifested by enhanced vascular permeability, increased lung edema, neutrophil accumulation, and worsened lung function. In addition to the unbalanced ACE/AT_1_R signaling, our group recently showed that lack of pulmonary ACE2 also results in impaired inactivation of des-Arg^9^-bradykinin, leading to enhanced bradykinin B1 receptor stimulation and exacerbated lung inflammation in response to bacterial endotoxin challenge^43^.

A hallmark and long-term consequence of ARDS is decreased lung compliance due to lung fibrosis. Using the bleomycin lung fibrosis model, Rey-Parra et al.^44^ observed that male *Ace2*^*−/y*^ mice exhibited a more significant decline in exercise capacity, worse lung function, and exacerbated lung fibrosis compared with WT. In comparison to males (*Ace2*^*−/y*^*)*, female (*Ace2*^*−/−*^) mice exposed to bleomycin harbored better lung function and architecture and reduced collagen deposition^44^, suggesting a sex dichotomy consistent with current reports on COVID-19 prevalence in men versus women.

In terms of viral lung infection, mice lacking ACE2 suffer more severe lung damage compared with WT mice following Influenza A H5N1 and H7N9 infection^45,46^. Similar findings were reported following infection with respiratory syncytial virus^47^. Taken together, the above observations suggest that this strain of mice could be useful to study SARS-CoV-2 infection-induced ALI/ARDS.

### United States

Another popular ACE2 KO model was reported by Gurley et al.^15^ in 2006 and was generated in collaboration with Millenium Pharmaceuticals Inc., using a similar strategy as used by Crackower et al. (Fig. 2)^14^. Chimeric mice were originally backcrossed onto C57BL/6J or 129/SvEv background. A lack of ACE2 mRNA transcript or protein was confirmed in the kidneys of *Ace2*^*−/y*^ animals. These ACE2 KO mice were reported to be fertile, lacked any gross anatomical or structural abnormalities, and had a normal lifespan^15^. However, in our hands and others^48,49^, these mice were found to be smaller and slower to develop compared with WT C57BL/6J. Gestation was also negatively affected as *Ace2*^*−/−*^ dams presented with impaired gestational weight gain, fetal growth restriction, and increased pup resorption that was associated with increased Ang-II levels in the placenta, placental hypoxia, and reduced umbilical blood flow velocity^50,51^. Similar to the ACE2 KO line generated by Yamamoto et al.^17^, these *Ace2*^*−/y*^ mice on mixed genetic backgrounds were found to lack the cardiac phenotype originally reported by Crackower et al.^14^. Instead, a mild elevation of systolic blood pressure (BP) was shown, which was conserved after backcrossing onto the C57BL/6J but not the 129/SvEv background^15^. Nevertheless, these mice showed enhanced hypertension^15^ and myocardial dysfunction^52^ after subcutaneous infusion of high levels of Ang-II. Similarly, increased systolic BP was observed in these ACE2 KO mice following high-fat-diet feeding^53^ or induction of type 1 diabetes^48^. Additional studies using this model revealed that *Ace2* deletion resulted in elevated fasting blood glucose levels^49^, glucose intolerance^49^, reduced glucose-stimulated insulin secretion and β-cells mass^54^, increased susceptibility to atherosclerotic plaques^55^, autonomic dysfunction^56^, increased oxidative stress^56^, and abdominal aortic aneurysms^57^.

As part of a systematic phenotyping project including 472 mouse KO lines, another ACE2 KO was generated by Genentech-Lexicon using homologous recombination of exon 1 (Fig. 2)^16^. Unlike other ACE2 KO, prenatal lethality was observed in these *Ace2*^*−/−*^ homozygous mutants. While details are lacking, a systematic screening indicated that the mutation affected multiple organ systems, including the heart, brain, and eyes. In males, a slightly smaller size and reduced weight gain were observed at baseline^58^. When subjected to a liver injury protocol, these mice exhibited enhanced hepatic inflammation and sensitivity to liver fibrosis^58^. When exposed to cigarette smoke^59^ or particulate matter^60^, these ACE2 KO mice showed a more severe reduction in body weight, greater increases in resting respiratory rate, pulmonary inflammatory response, and bronchial hyperplasia when compared with WT controls. This line has been cryo-archived at the Mutant Mouse Resource and Research Center (# 031665-UCD).

### Japan

Instead of targeting the enzyme’s catalytic site, Yamamoto et al.^17^ directed their neomycin cassette to exon 3, thus removing codons 116–147, including the splice donor and acceptor sites (Fig. 2). Mice were bred on a C57BL6J background, and the lack of ACE2 was confirmed by western blotting and real-time qPCR. The authors failed to see the altered cardiac function and hypertrophy reported by Crackower et al.^14^, although a follow-up study^61^ identified postnatal cardiac hypertrophy in these mice. Reduction in cardiac contractility and dilatation of the left ventricle were observed in response to pressure overload^17^. In this model, the elevation of cardiac and serum Ang-II levels and enhanced AT_1_R downstream signaling, which was blocked by the AT_1_R blocker candesartan, suggest that the lack of clearance of Ang-II by ACE2 contributed to the disease^17^. Although body weight, mean BP, kidney weight, serum Ang-II, and creatinine were not different between *Ace2*^*−/y*^ and WT controls, kidney Ang-II levels showed a threefold increase at baseline, and induction of type 1 diabetes led to an earlier onset of albuminuria and a more severe glomerular/tubulo-interstitial damage in *Ace2*^*−/y*^ mice^62^.

Additional studies in this ACE2 KO model revealed increased cuff-induced neointimal formation^63^, glucose intolerance and insulin resistance^64^, reduced glucose transporter (GLUT) 4 and myocyte-specific enhancer factor 2A expression in the muscle^64^, atherosclerotic lesions^65^, impaired cognitive function with associated increases in AT_1_R expression and oxidative stress in the hippocampus^66^. Enhanced expression of sarcopenia-associated genes was also observed, and Ang-(1–7) infusion improved the lack of muscle strength and bone loss in these *Ace2*^−/y^ mice^67^. While the metabolic disturbances due to ACE2 deficiency are consistent with COVID-19 reports^68^, the impact of SARS-CoV-2 infection on cognitive deficit and muscle strength in these patients is not yet known.

### China

Targeted homologous recombination (−2000 to +489 bp DNA fragment of *Ace2* gene) in D3 embryonic stem cells was used by Qin et al.^18^ (Fig. 2) to generate this ACE2 KO model, and the deletion was confirmed by western blot analysis. Impaired first phase insulin secretion, enhanced glucose sensitivity, reduction in liver expression of GLUT2, enhanced oxidative stress, and dedifferentiated β-cells were reported in this ACE2 KO line, although baseline body weight, fasting blood glucose, and insulin levels were not altered^69–71^. *Ace2*^*−/y*^ subjected to limb ischemia–reperfusion protocol presented with enhanced remote renal and lung injury as well as increased mortality^72,73^. Lack of ACE2 was also shown to induce more severe acute pancreatitis^74^. In addition, *Ace2*^*−/y*^ mice exhibited enhanced response to lipopolysaccharide-induced lung injury, and transplantation of mesenchymal stem cells overexpressing ACE2 rescued lipopolysaccharide-induced lung damage^75^. This model is available from the Institute of Laboratory Animal Sciences at the Chinese Academy of Medical Sciences and Peking Union Medical College (Beijing, China).

Liu et al.^19^ recently described a mouse line generated using CRISPR/Cas9 technology, with the most extensive *Ace2* gene deletion so far (Fig. 2). The deletion spanning exons 3–18 was confirmed at both mRNA and protein levels. No phenotypical data are currently available for these mice. CRISPR/Cas9 gene targeting technology was also used to generate a frameshift mutation in the *Ace2* gene at an unknown location, resulting in a novel ACE2 KO line^20^, which showed aggravated PH induced by SU5416/hypoxia treatment, accompanied by markedly reduced endothelial nitric oxide (NO) synthase phosphorylation. Another model designed using CRISPR/Cas9 is available on a C57BL6J background (B6/JGpt-*Ace2*^*em1Cd*^/Gpt) from the National Resource Center of Model Mice (NRCMM; www.nrcmm.cn) and Model Animal Research Center of Nanjing University (MARC).

An outbred ACE2 KO mouse line was generated using TALEN-induced double-strand breaks targeting exon 2 of the *Ace2* locus immediately downstream of the start codon (Fig. 2), resulting in *Ace2* frameshift mutations^21^. The generated *Ace2*^*−/y*^ mice, on a Kunming background, displayed increased susceptibility to dextran sodium sulfate-induced colitis, enhanced histological damage in the colon, and up-regulated expression of inflammatory cytokines^21^.

### Summary of global ACE2 KO models

Although the existing global ACE2 KO models described above exhibit some differences at baseline (body weight, BP, cardiac phenotype, etc.), their responses to cardiometabolic challenges or inflammatory insults are largely consistent, highlighting the protective roles of ACE2 in cardiovascular, pulmonary, and metabolic diseases. SARS-CoV-2 hijacks cellular ACE2 and disrupts these protective effects, which could explain the increased prevalence and severity of COVID-19 in patients with hypertension, diabetes, and metabolic diseases who already suffer from increased Ang-II/AT_1_R signaling and/or reduced ACE2 function^68^.

## Conditional ACE2 KO models

Our group recently reported the development of a floxed *Ace2* model amenable for deletion in specific cells or tissues using Cre-LoxP technology^76^. These mice were generated at the Pennington Biomedical Research Center Transgenic Facility (Louisiana State University) using embryonic stem cells obtained from the Knockout Mouse Project, where LoxP sites flank exon 4 (Fig. 2). A similar model has been reported by Shoemaker et al.^77^. Using CRISPR/Cas9, another conditional ACE2 KO model is available on the C57BL6J background (B6/JGpt-*Ace2*^*em1Cflox*^/Gpt) from the NRCMM and MARC, but no details are listed about the construct. Conditional ACE2 KO models and associated phenotypes are listed in Table 2.

### ACE2 deletion on neurons

ACE2 has previously been reported to contribute to the prevention of neurogenic hypertension by transforming Ang-II into Ang-(1–7), thus limiting oxidative stress, improving cardiac baroreflex, and autonomic function^56,78^. Using synapsin1-cre mice, our group investigated the role of ACE2 in a specific population of hypothalamic neurons involved in BP regulation^76^. Selective deletion of *Ace2* from neurons was associated with a significant reduction of ACE2 activity in the brain. Patch-clamp performed on hypothalamic neurons projecting to the kidney revealed a reduced inhibitory postsynaptic current suggesting that ACE2 supports the inhibitory tone to these neurons. This was further supported by the identification of ACE2 on GABAergic neurons^76^. This model might be relevant to the study of SARS-CoV-2 penetrance and tropism to the central nervous system^79^.

### ACE2 deletion on lung epithelial cells

Using the same *Ace2* floxed line^76^, we generated a mouse model in which the *Ace2* gene was specifically excised from Forkhead box protein J1 (Foxj1)^+^ lung epithelial cells (*Ace2*^*ΔFoxj1*^) by cross-breeding the floxed mice with Foxj1^cre^ mice^42^. The progeny was healthy, fertile, displayed no distinct lung phenotype, and reproduced at expected Mendelian ratios. As expected, these mice exhibited reduced ACE2 expression and activity in the lung. When subjected to a *Pseudomonas aeruginosa* bacterial pneumonia model, *Ace2*^*ΔFoxj1*^ mice demonstrated more significant weight loss, increased neutrophil infiltration, exaggerated lung injury, and inflammation in comparison to their WT counterparts^42^. These results indicate that pulmonary epithelial ACE2 plays a significant role in modulating lung inflammation in response to pathogenic insults.

### ACE2 deletion on adipocytes

ACE2 contributes to sex differences in the development of obesity and hypertension, as estrogens are thought to increase ACE2 expression on adipocytes of obese females, thus attenuating the metabolic and hemodynamic phenotypes^53^. To investigate the protective role of ACE2 in the adipose tissue, Shoemaker et al.^77^ bred *Ace2*^*fl/y*^ harboring Cre recombinase under the control of the adipocyte-specific promoter adiponectin with homozygous floxed females (*Ace2*^*fl/fl*^). ACE2 mRNA was reduced by ~50% in adipose tissue while unaffected in other tissues. Although the development of obesity was not affected in females lacking ACE2 on adipocytes, systolic BP and the acute pressor response to Ang-II were increased in these animals^77^. Together, these data show that ACE2 expression on adipocytes regulates BP in a sex-dependent manner and that SARS-CoV-2 induced alteration of ACE2 function could aggravate cardiovascular dysfunction in obese individuals.

## ACE2 KI models

**Fig. 3:**
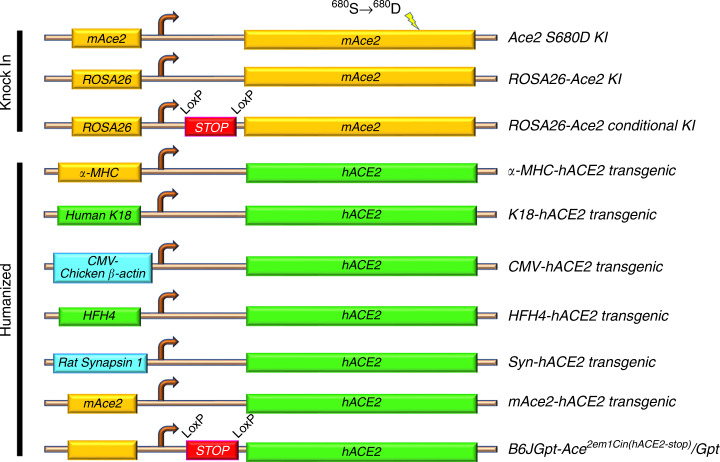
Expression of ACE2 in transgenic and KI mice. Schematic of the different gain-of-function models driven by specific mouse (gold), human (green), or other (blue) promoters for ubiquitous or targeted ACE2 overexpression. ROSA26: locus used for constitutive and ubiquitous gene expression; K18: cytokeratin 18 promoter targeting epithelial cells; CMV: cytomegalovirus promoter for constitutive expression; HFH4: lung ciliated epithelial cell-specific promoter; Syn: synapsin1 promoter for neuronal expression; α-MHC: α-myosin heavy chain for cardiac-specific expression. LoxP: sites for cre-recombinase-mediated excision of the stop codon.

These models are particularly useful for gain-of-function studies. Here, the Ace2 gene has either been mutated or its expression controlled by a specific promoter (Fig. 3 and Table 3).

### Ace2 S680D KI

While investigating the role of ACE2 in the pathogenesis of PH, Zhang et al.^20^ found that Serine (Ser) phosphorylation at position 680 of the ACE2 protein by AMP-activated protein kinase leads to enhanced stability of ACE2, thereby resulting in increased Ang-(1–7) and enhanced NO bioavailability in cultured endothelial cells. To address the translational implication of this observation in PH and other cardiovascular diseases in vivo, the authors created an *Ace2* S680D KI mouse line using CRISPR/Cas9 genomic editing to replace serine (S) with aspartic acid (D), resulting in a gain-of function mutation due to reduced ubiquitin-mediated degradation of *Ace2* S680D mutant product^20^. When subjected to SU5416/hypoxia–reoxygenation, *Ace2* S680D KI mice exhibited reduced right ventricular systolic pressure and reduced Fulton index (a measure of right ventricular hypertrophy) compared with WT mice, although systolic BP did not differ. In addition, pulmonary artery muscularization, luminal narrowing, and abnormal vascularization observed in WT mice following SU5416/hypoxia–reoxygenation were largely absent in *Ace2* S680D KI mice^20^. These observations suggest that a single gain-of-function mutation at *Ace2* ^680^Ser is protective against the development of PH. It is unclear whether mutation of ACE2 in these mice would affect infection with SARS-CoV-2.

### ROSA26-Ace2 KI

In this inbred line, maintained on a mixed C57BL/6 and 129/Sv background, the mouse *Ace2* gene is expressed under the control of the endogenous ROSA26 promoter^80^. These *Ace2* KI mice exhibit increased ACE2 mRNA expression ranging from fivefold in the kidney, 60-fold in the heart, to 230-fold in the hypothalamus^80,81^. ACE2 activity in the plasma was increased by fourfold, although Ang-(1–7) levels only increased by 50%, while Ang-II levels were not significantly affected^80,81^. Despite these massive increases in ACE2 expression and activity, cardiac and hemodynamic parameters were not altered under baseline conditions. Following myocardial infarction, however, these mice were shown to have reduced infarct size and associated inflammation^81^. Ang-(1–7)-mediated reduction in anxiety and plasma corticosterone levels were also noted in these animals^80^, supporting an anxiolytic function of the central ACE2/Ang-(1–7)/MasR signaling. A similar model generated using CRISPR/Cas9 is available on a C57BL/6 background (B6JGpt-*Ace2*^em1Cin(*hACE2*-stop)^/Gpt) from the NRCMM and MARC.

### ROSA26-Ace2 conditional KI

By inserting a STOP codon flanked by LoxP sites between the ROSA26 promoter and the *Ace2* gene in the above *Ace2* KI mice, Wang et al.^82^ generated a new line amenable to tissue-specific overexpression of mouse ACE2. Selective expression in corticotropin-releasing hormone (CRH)-positive cells was achieved by breeding with a CRH-cre mouse. Increased ACE2 mRNA expression was verified in CRH-producing cells of the paraventricular nucleus of the hypothalamus using in situ hybridization. CRH-*Ace2* KI mice were shown to have reduced anxiety-like behavior and reduced plasma cortisol levels following restraint stress^82^, further confirming the role of central ACE2 in the regulation of hypothalamic–pituitary–adrenal axis.

Using the above ROSA26-*Ace2* conditional KI^82^, the same group initiated targeting of ACE2 expression to the microvilli of the brush border of the epithelium lining of the gut using a villin-cre mouse to generate Villin-*Ace2* KI (M.K. Raizada, Personal communication, 2020), but no phenotype has been reported yet.

## Humanized ACE2 models

In these models, the human *ACE2* gene (*hACE2*) has been placed under control of a promoter of choice to induce expression in a specific tissue or cell type in mice (Fig. 3 and Table 4). These strains are readily susceptible to SARS-CoV-2 infection and would be invaluable for studies of the pathogenesis and antiviral therapies for COVID-19.

### αMHC-hACE2

Following their co-discovery of ACE2^1^, Donoghue et al.^83^ made the first ACE2-humanized mouse by injecting a 9.4 kb fragment containing the *hACE2* cDNA under the control of a mouse cardiac α-myosin heavy chain (αMHC) promoter, followed by a human growth hormone polyA sequence. These mice were generated on the FVB background. Specific expression in the heart was confirmed by western blotting, and ACE2 activity in the serum was enhanced by 10-fold. BP was reported to be low, but surprisingly, high mortality was observed with progressive cardiac conduction disturbances, A-V block, and lethal ventricular arrhythmias. This phenotype was associated with down-regulation of connexin 40 and 43 in the heart and presumably adverse gap junction remodeling. This deleterious cardiac phenotype was not observed in global ROSA26*-Ace2* KI mice^81^. It is our understanding that due to its lethality, this model is no longer available.

### K18-hACE2

As we discussed previously, murine ACE2 does not support SARS-CoV or SARS-CoV-2 binding as efficiently as human ACE2, and mice infected with SARS-CoV rarely showed significant clinical disease. To tackle this issue, McCray et al.^84^ generated a transgenic mouse in which expression of *hACE2* was driven by the human cytokeratin 18 (K18) promoter targeting epithelial cells (K18-*hACE2* mice). Specifically, the transgene contains the full-length *hACE2* coding cDNA under the control of the K18 promoter followed by the polyA signal of the human K18 gene. This transgene was injected into pronuclei of fertilized (C57BL/6JXSJL/J) F2 mouse eggs to generate transgenic embryos, and the progenies were backcrossed two to three times onto a C57BL/6 background. The K18 promoter confers efficient transgene expression in airway epithelial cells (but not in alveolar epithelia), as well as in epithelia of other internal organs, including the liver, kidney, and gastrointestinal tract. As a confirmation of such a K18-directed expression pattern, human ACE2 mRNA was detected in several tissues, including the lung, colon, liver, and kidney of the transgenic mice.

Following intranasal inoculation of SARS-CoV at 2.3 × 10^4^ plaque-forming unit, K18-*hACE2* mice suffered rapid weight loss with lethargy and labored breathing, and all mice succumbed to infection by 7 days post infection (dpi)^84^. By contrast, infection of non-transgenic littermates resulted in no mortality or clinical disease. Importantly, viral titers were 3 log units higher in K18-*hACE2* mice than in WT mice, suggesting enhanced viral replication in the K18-*hACE2* mice. The 50% lethal dose of SARS-CoV for K18-*hACE2* mice was less than 230 plaque-forming unit after intranasal inoculation. In addition, widespread inflammatory infiltrates, increased inflammatory cell margination through vessels, more epithelial cell sloughing, and more signs of lung injury were observed in K18-*hACE2* mice compared with their non-transgenic littermates, reminiscent of the pulmonary findings described for SARS patients. In K18-*hACE2* mice, however, no evidence of diffuse alveolar damage commonly found in ARDS was observed, possibly due to the absence of *hACE2* expression in the alveolar epithelium. Instead, patchy, intense neutrophilic infiltration was noted in the lungs of some K18-*hACE2* mice, leading to airway obstruction, degenerate neutrophil aggregates, foci of necrotizing bronchopneumonia, and alveolar flooding with seroproteinaceous fluid. In addition, proinflammatory cytokines and chemokines including interferon-γ, CXCL9, CXCL10, CCL2, and CCL7 were elevated in the lungs of SARS-CoV-infected K18-*hACE2* mice in comparison to WT mice at 2 dpi. Remarkably, interferon-α/β mRNA was not detected in the infected lungs of K18-*hACE2* mice, consistent with the observation that SARS-CoV does not induce efficient type 1 interferon response^85,86^. In a follow-up study using the same line, Netland et al.^87^ reported that intranasal inoculation resulted in some viruses entering the brain via the olfactory bulb, leading to rapid, trans-neuronal spread to connected areas of the brain and neuronal cell death. This finding suggests that neurons are a highly susceptible target for SARS-CoV, which is consistent with brain infections observed in SARS patients^88^. In addition, Netland et al.^89^ used K18-*hACE2* mice to test and validate immunization efficacy using an attenuated SARS-CoV with deleted envelope protein to protect against SARS. Taken together, K18-*hACE2* mice infected with SARS-CoV recapitulated most of the clinical findings in SARS patients and would be a suitable model for COVID-19 research. Indeed, a recent study showed that infection with SARS-CoV-2 in the K18-*hACE2* mice resulted in high viral titers in the lungs with altered lung histology, interstitial inflammatory cell infiltration, and alveolar septal thickening^90^. This mouse line is available at The Jackson Laboratory (Stock No: 034860).

### CMV-hACE2

As McCray et al.^84^ revealed their strain of K18-*hACE2* transgenic mice, Tseng et al.^91^ reported another humanized mouse line driven by a composite global promoter consisting of the cytomegalovirus (CMV) immediate-early enhancer and the chicken β-actin promoter. Transgenic mice expressing *hACE2* were generated by the conventional technique of microinjecting the expression cassette into pronuclei of zygotes from the intercross of (C57BL/6J XC3H/HeJ) F1 parents, which were then backcrossed two to three times onto either a C57BL/6 or BALB/c background. A total of five founder lines were established^91^ and *hACE2* expression levels in different founder lines strongly correlated with the severity and outcome of SARS-CoV infection^91,92^. When subjected to SARS-CoV intranasal inoculation, some lines demonstrated both lung and brain infection, whereas others exhibited lung-restricted infection. Intranasal inoculation in high *hACE2* expressing mice resulted in clinical manifestations including ruffled fur, lethargy, rapid and shallow breathing, and persistent weight loss (up to 35–40% in some mice).

In CMV-*hACE2* mice (high expressers), mortality began at 4 dpi and reached 100% by 8 dpi, whereas SARS-CoV-infected WT mice continued to thrive throughout the entire course of study^91^. Similar to what McCray et al.^84^ reported, the lungs and the brain were the major sites of viral replication. The viral titer in the lungs peaked within 1–2 dpi, whereas in the brain, the virus was first detected at 2 dpi, reaching high titer at 3 dpi which was then maintained until death, contributing to the high mortality in SARS-CoV-infected transgenic mice. Tseng et al.^91^ further revealed that although SARS-CoV infection failed to induce cytokine production in WT mice, elevated levels of interleukin-1β (IL-1β), IL-12p40/p70, CXCL1, RANTES, and monocyte chemoattractant protein-1 expression were readily detected in the lungs of transgenic mice. Moreover, highly elevated levels of IL-6, IL-12p40, granulocyte colony-stimulating factor, CXCL1, macrophage inflammatory protein-1α, and monocyte chemoattractant protein-1 were detected at day 3 in the brain of these transgenic mice, concomitant with the increase in viral titer.

### HFH4-hACE2

Menachery et al.^93^ reported a transgenic mouse line with airway targeted overexpression of *hACE2*. These mice were generated by microinjection of fertilized C3H × C57BL/6 (C3B6) F1 hybrid oocytes with an expression cassette consisting of the hepatocyte nuclear factor-3/forkhead homolog 4 (HFH4)/Foxj1 lung ciliated epithelial cell-specific promoter elements and the coding region of *hACE2* cDNA in a pTG1 vector. Founder mice were then crossed to C3B6 to produce *hACE2* transgenic mice. Menachery et al.^93^ revealed that although robust *hACE2* gene expression was observed in the lung, other tissues of the transgenic mice including the brain, liver, kidney, and gastrointestinal tract displayed varying levels of *hACE2* expression, indicating greater tissue distribution of HFH4-mediated expression than initially expected. The authors challenged these mice with SARS-CoV Urbani strain by nasal inoculation and found that HFH4*-hACE2* mice exhibited rapid weight loss and death between days 4 and 5, with robust viral replication detected in both the lungs and brain. Further therapeutics testing against coronavirus infection using this model revealed that a SARS-CoV monoclonal antibody protected these mice from lethal challenges of both SARS-CoV Urbani and the SARS-like WIV1-CoV^93^. These mice have been transferred to the Mutant Mouse Resource and Research Center (#066719-UNC) for archival and distribution.

### Syn-hACE2

The same 4.4 kb fusion transgene used for the K18-*hACE2* mice, containing the full-length *hACE2* cDNA, here driven by a rat synapsin1 promoter and followed by a polyA tail, was microinjected into fertilized C57BL/6JxSJL/J (B6SJLF2) mouse embryos at the University of Iowa Transgenic Animal Facilities^94^. From the founders generated, line 10 expressed the highest levels of transgene in the brain and was selected for further development. The specificity of the transgene was confirmed by real-time qPCR, and distribution of human ACE2 was detected throughout the brain using immunohistochemistry. Baseline Ang-II levels and AT_1_R expression were found to be reduced in the brainstem and hypothalamus, notably in the nucleus of tractus solitarius, whereas AT_2_R and MasR were up-regulated. Immunoreactivity to both neuronal and endothelial NO synthases, as well as the phosphorylated form of endothelial NO synthase, was enhanced in the brain of these mice with increased local NO levels, consistent with increased Ang-(1–7)/MasR signaling. However, baseline BP, heart rate, baroreflex, and autonomic functions remained normal^94^. When infused with Ang-II, Syn-*hACE2* mice presented with a blunted hypertension and a drastically reduced water intake compared with control littermates. Ang-II-induced hypertension was restored following blockade of MasR, confirming the critical role of Ang-(1–7)/MasR signaling in the beneficial effects of ACE2^94^. In addition, Ang-II-infused Syn-*hACE2* mice presented with reduced cardiac hypertrophy and enhanced *hACE2* expression was shown to counter ADAM17-mediated shedding of the enzyme from the plasma membrane, resulting in enhanced clearance of Ang-II and reduced AT_1_R signaling. Following coronary artery ligation to induce heart failure, Syn-*hACE2* mice exhibited reduced renal sympathetic nerve activity and lower urinary norepinephrine levels compared with control mice, supporting the beneficial role of neuronal ACE2 in countering enhanced sympathetic activity^95^. This finding was confirmed in another study where Ang-II infusion also resulted in reduced norepinephrine levels and cardiac hypertrophy in Syn-*hACE2* mice^96^.

Although Syn*-hACE2* mice have not been used for SARS research, it is potentially a useful model since the brain is a major site of coronavirus replication^79,84^. These mice have been deposited at The Jackson Laboratory (Stock No. 034899).

### mAce2-hACE2

Another humanized ACE2 transgenic mouse line was generated by Yang et al.^97^, in which *hACE2* expression was driven by the mouse *Ace2* promoter (*mAce2*). Hemizygous animals did not show any adverse phenotype, and *hACE2* mRNA was detected by real-time qPCR in the lung, heart, kidney, and intestine of F1 offspring. A similar protein expression pattern was confirmed by western blotting. At days 3 and 7 post-inoculation, SARS-CoV replicated more efficiently in the lungs of transgenic mice than in those of WT mice. In addition, transgenic mice had more severe pulmonary lesions, including interstitial hyperemia and hemorrhage, monocytic and lymphocytic infiltration, protein exudation, and alveolar epithelial cell proliferation and desquamation. Vasculitis, degeneration, and necrosis were also found in the extrapulmonary organs of transgenic mice, and viral antigen was found in the brain. The above findings indicate increased susceptibility of the transgenic mice to SARS-CoV than WT controls, which was associated with severe pathologic changes that resembled human SARS infection.

This *mAce2-hACE2* line has recently been adopted in the study of SARS-CoV-2^98^. Like SARS-CoV, SARS-CoV-2 infected *mAce2-hACE2* mice exhibited greater weight loss and higher viral replication in the lungs compared with WT animals. The typical histopathology included interstitial pneumonia with infiltration of inflammatory cells, with viral antigens (spike-1 protein) detected in the bronchial epithelial cells, alveolar macrophages, and alveolar epithelia. In contrast to SARS-CoV, apart from the intestine, SARS-CoV-2 viral loads in extrapulmonary organs including the brain, kidney, heart, and liver were low or undetectable. This finding is consistent with the lower mortality rates associated with SARS-CoV-2 infection in patients. This timely study confirms the value of *mAce2-hACE2* mice in SARS-CoV-2 research, especially in search for therapeutics and vaccines for COVID-19. This mouse line has also been used to study remote organ injury following hindlimb ischemia–reperfusion. In contrast to *Ace2*^*−/y*^ mice which exhibited enhanced remote renal and lung injury following hindlimb ischemia–reperfusion, *mAce2-hACE2* mice appeared to be protected from these effects^72,73^.

This model originated from the Institute of Laboratory Animal Science, Peking Union Medical College (Beijing, PR China) but availability is currently unknown.

### hACE2 KI

Using CRISPR/Cas9 technology and similar to the strategy described by Qi et al.^81^, a *hACE2* KI line was generated to allow targeted expression of *hACE2* in a specific tissue or cell type. These mice are available live from the NRCMM and MARC (strain #9340).

## Conclusion and future directions

Over the past two decades, numerous genetically engineered mouse models have been generated to understand the role of ACE2 with regards to cardiovascular, pulmonary and infectious diseases. With the emergence of COVID-19, the interest in ACE2 models has intensified. While ACE2 serves as the host entry receptor for SARS-CoV-2, alteration of the renin–angiotensin system resulting from impaired ACE2 function following coronavirus infection and how that contributes to the development of COVID-19 cannot be overemphasized. The ACE2 mouse models discussed in this review provide valuable resources to study the mechanisms of coronavirus infection, including in at risk patients with cardiovascular and metabolic diseases, as well as to identify therapeutic strategies to combat the COVID-19 pandemic.

ACE2 plays pleiotropic roles in diverse biological processes, and the COVID-19 pandemic also highlighted our lack of understanding of ACE2 function in areas such as coagulation and neurological functions. Although platelets do not express ACE2^99^, they do express that MasR and the ACE2/Ang-(1–7)/MasR axis exerts important anti-thrombotic effects^100^. Similarly, the role of ACE2 in cognitive function remains largely unexplored. Strategies to directly augment Ang-(1–7)/MasR signaling in the face of diminished ACE2 function in COVID-19 patients may prove to be effective treatment options. Finally, our understanding of ACE2 function outside the renin–angiotensin system is very limited. For instance, as we reported previously, ACE2 is critical to inactivate des-Arg^9^-bradykinin and deficiency of pulmonary ACE2, which has been proposed in COVID-19 patients, could lead to pulmonary angioedema and exacerbated lung inflammation. Alteration of bradykinin metabolism due to impaired ACE2 function could also contribute to the pathogenesis of COVID-19.
